# Admission CALLY Index and Angiographic No-Reflow After Primary PCI in a Retrospective STEMI Cohort

**DOI:** 10.3390/jcm15218724

**Published:** 2026-11-08

**Authors:** Fahrettin Turna, Emre Eynel, Muhammet Taşdemir, Arslan Nyyazov, Şevval Özdemir, Mehmet Şirin Yıldız, Emirhan Hancıoğlu, Yusuf Can, İbrahim Kocayiğit

**Affiliations:** 1 Department of Cardiology, Sakarya University Training and Research Hospital, Sakarya 54100, Turkey; fturna_53@hotmail.com (F.T.); emreeynel@gmail.com (E.E.); muhammet.75256@gmail.com (M.T.); 2 Department of Cardiology, Sakarya University School of Medicine, Sakarya 54100, Turkey; niyazzov_93@list.ru (A.N.); ozdemir_sevval@hotmail.com (Ş.Ö.); yusufcan@sakarya.edu.tr (Y.C.); 3 Department of Cardiology, Tunceli State Hospital, Tunceli 62000, Turkey; mehmet_sirin65@hotmail.com; 4 Department of Cardiology, Medipol University School of Medicine, İstanbul 34083, Turkey; hanciogluemir@gmail.com

**Keywords:** CALLY index, no-reflow, ST-segment elevation myocardial infarction, primary percutaneous coronary intervention, inflammation, nutritional status

## Abstract

Background/Objectives: The CALLY index draws on three admission tests—C-reactive protein, albumin, and lymphocyte count. We investigated whether it was associated with angiographic no-reflow after primary percutaneous coronary intervention (PCI) in patients with ST-segment elevation myocardial infarction (STEMI). Methods: We reviewed 1245 patients treated at one center between January 2024 and January 2026. Logistic regression accounted for clinical risk factors, baseline Thrombolysis in Myocardial Infarction (TIMI) flow, culprit artery, thrombus grade 5, Killip class at the start of PCI, and door-to-balloon time. We also tested alternative endpoint definitions, assessed influential observations, and compared correlated receiver operating characteristic (ROC) curves. Results: No-reflow occurred in 206 patients (16.5%). CALLY was lower in this group than in the reflow group (median 1.11 [0.39–1.92] vs. 1.33 [0.73–2.11]). Each one-unit increase in ln(CALLY) was associated with lower odds of no-reflow after adjustment (odds ratio [OR] 0.800, 95% confidence interval [CI] 0.693–0.923; *p* = 0.0023). The association remained when the endpoint was limited to impaired final angiographic reperfusion (OR 0.832, 0.717–0.965; *p* = 0.015). Discrimination by CALLY alone was poor (area under the curve [AUC] 0.578, 0.534–0.623); it was not significantly better than its components or the other indices studied. At a displayed threshold of ≤0.905, positive and negative predictive values were 22.8% and 86.8%. The association weakened after removal of the upper and lower 1% of values (*p* = 0.063). Conclusions: Admission CALLY was associated with recorded angiographic no-reflow, even after broader adjustment. Its weak discrimination and small incremental contribution, however, do not support use for individual risk classification without further study.

## 1. Introduction

Primary PCI restores epicardial flow in most patients presenting with ST-segment elevation myocardial infarction (STEMI), yet myocardial perfusion may remain poor despite an open infarct-related artery [1]. Once residual obstruction, dissection, spasm, and other mechanical causes have been excluded, this finding is generally described as angiographic no-reflow. Thrombolysis in Myocardial Infarction (TIMI) flow and myocardial blush offer practical angiographic assessments, but neither is a direct measure of microvascular obstruction. Patients with no-reflow tend to have more extensive myocardial injury and worse clinical outcomes [2].

Inflammation is one of several processes involved in reperfusion injury after STEMI [3]. Laboratory indices related to inflammation have also been studied in other PCI settings, such as saphenous vein graft intervention and restenosis [4,5], although those findings cannot be transferred directly to STEMI no-reflow. More relevant studies have examined inflammatory blood-cell counts and albumin-related indices after primary PCI [6,7,8,9]. CALLY incorporates C-reactive protein (CRP), albumin, and lymphocyte count; it falls when CRP rises or either of the other two measurements falls. The index has already been studied in STEMI, including in relation to no-reflow and contrast-associated acute kidney injury [10,11,12]. What remains uncertain is whether CALLY retains an association with no-reflow after accounting for the initial angiogram and clinical severity, and whether the combined index offers more information than the individual measurements. We addressed these questions in a retrospective cohort, with additional analyses of endpoint definition and discrimination.

## 2. Materials and Methods

### 2.1. Study Design and Population

We retrospectively reviewed patients who underwent primary PCI for STEMI at Sakarya University Training and Research Hospital from 1 January 2024 through 1 January 2026. This was a single-center study; both patient care and data collection took place at that hospital. Approval was obtained from the Non-Interventional Clinical Research Ethics Committee of Istanbul Medipol University on 27 August 2026 (decision no. 1310). The study followed the Declaration of Helsinki. Before ethics approval, written permission to use the hospital records was obtained from the source institution and the head of the clinical department; these permissions were included in the ethics application. The ethics approval specified that individual patient consent was not required for this retrospective study.

We screened admissions for patients aged 18 years or older who underwent primary PCI for STEMI within the study period. STEMI was diagnosed from the clinical presentation, electrocardiogram, and cardiac biomarker results, in keeping with current diagnostic recommendations.

We excluded admissions without a confirmed STEMI diagnosis and patients with an admission estimated glomerular filtration rate (eGFR) below 30 mL/min/1.73 m^2^, maintenance dialysis, or a recorded inflammatory, infectious, hematological, or autoimmune disorder. These diagnoses were identified from hospital discharge summaries, with treatment information and hospital diagnostic codes also considered. They were clinical record-based exclusions. The available study documentation does not provide a complete disease-specific diagnostic or coding algorithm, so the application of each exclusion cannot be independently reproduced for all screened patients. Severe renal impairment was excluded because it can alter inflammatory measurements, albumin, and circulating lymphocyte counts, all of which contribute to CALLY. Inclusion also required the three admission laboratory measurements needed to calculate CALLY and angiograms adequate for TIMI flow and myocardial blush grade (MBG) assessment. Short angiographic acquisitions that did not permit blush assessment were excluded. Admission eGFR was recalculated using the race-free 2021 CKD-EPI creatinine equation with age and sex; earlier creatinine results were not available for this analysis.

**Figure 1:**
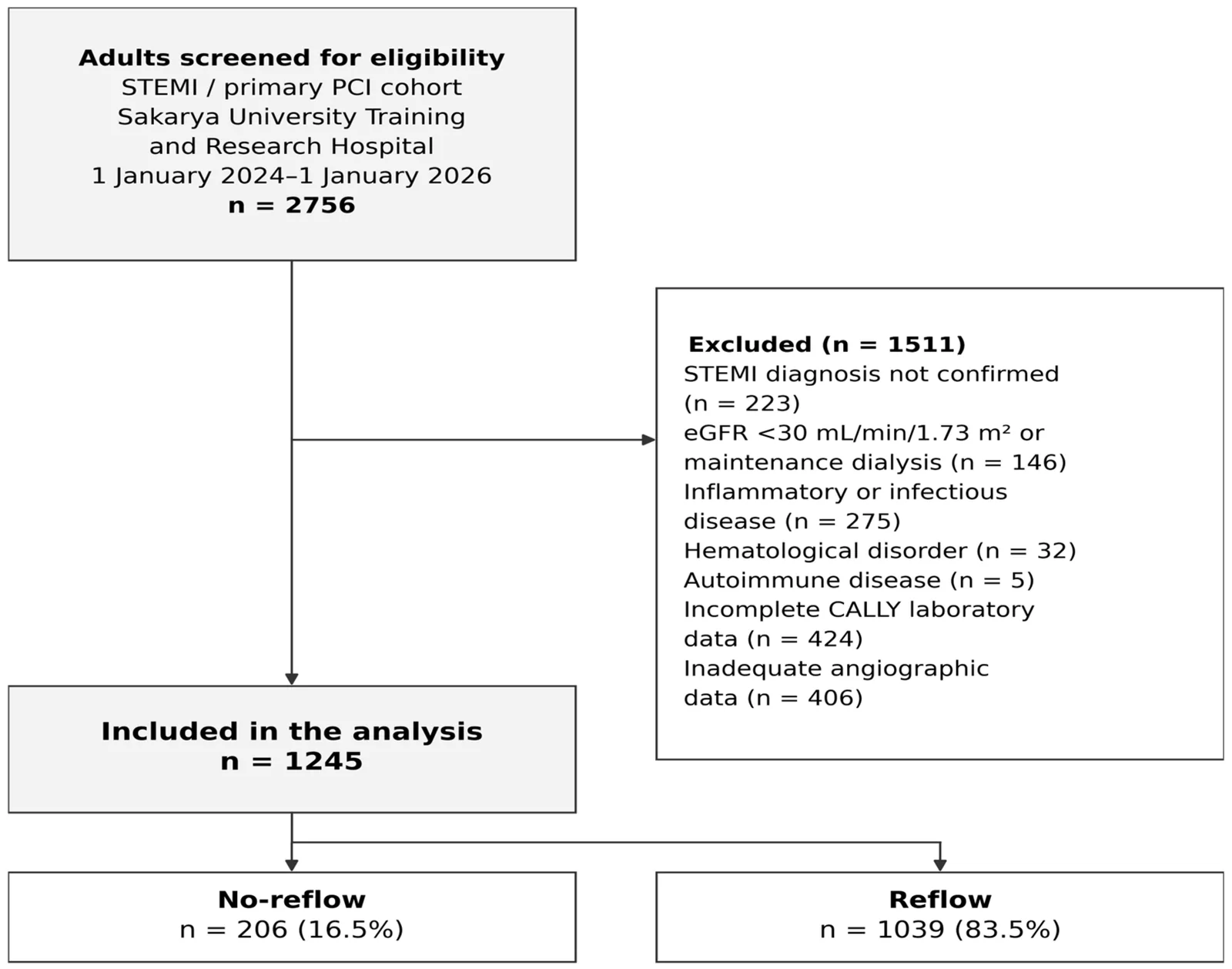
Screening flow and allocation of the 1245 patients according to angiographic no-reflow status. CALLY, C-reactive protein–albumin–lymphocyte index; eGFR, estimated glomerular filtration rate; PCI, percutaneous coronary intervention; STEMI, ST-segment elevation myocardial infarction.

Clinical history, cardiovascular risk factors, admission blood tests, and procedural and angiographic findings were extracted from hospital records and the catheterization database. Smoking was coded as current or former versus never. For hyperlipidemia and other history variables, we used the recorded entries; the analytic file could not distinguish an undocumented history from a negative entry. We checked the records for duplicates before establishing the final cohort of 1245 unique patients (Figure 1). The variables used in the regression analyses were complete, so imputation was not required.

Door-to-balloon time was measured in minutes, from registration in the emergency department to balloon inflation. Killip class was taken from the assessment at the start of PCI. Because very few patients were in classes III or IV, the principal model compared class I with classes II–IV; other codings were explored separately. Reliable symptom-to-reperfusion times and a separate preprocedural cardiac-arrest variable were not available.

In all, 54.8% of screened patients did not enter the analysis (Figure 1). Two major reasons were incomplete CALLY laboratory data (424 patients) and angiographic records insufficient for assessment (406 patients), including short acquisitions that did not permit reliable MBG grading. We lacked the clinical data needed to compare the included cohort with all those excluded at screening. We could not determine whether missing laboratory or angiographic information was related to clinical urgency, illness severity, or procedural complexity.

### 2.2. Laboratory Measurements and Calculation of the CALLY Index

Blood samples were obtained at admission, before PCI. Hematological tests were performed with an LH 780 analyzer (Beckman Coulter, Brea, CA, USA). Albumin was measured spectrophotometrically and standard CRP by immunoturbidimetry on an AU5800 chemistry analyzer (Beckman Coulter, Brea, CA, USA). We recorded blood counts in ×10^9^/L, albumin in g/L, CRP in mg/L, and creatinine and uric acid in mg/dL. CALLY was calculated as [albumin (g/L) × lymphocytes (×10^9^/L)]/[10 × CRP (mg/L)]. Because CALLY was positive in every patient, it could be log-transformed without an offset. Analyses used ln(CALLY) to express relative changes and lessen the influence of very high values. Time from symptom onset to sampling could not be determined.

We analyzed CRP values as recorded rather than replacing low results. A noticeable number of reports were exactly 3.22 mg/L, although smaller values also appeared in the dataset. Changes in assay kits or reporting limits might account for some of this clustering, but the records needed to confirm that explanation were unavailable. We therefore repeated the analysis after excluding values of exactly 3.22 mg/L and, separately, among patients with CRP > 3.34 mg/L. The latter was chosen for sensitivity analysis, not as a verified laboratory detection limit.

### 2.3. Coronary Angiography and Primary Percutaneous Coronary Intervention

Primary PCI and emergency angiography were carried out according to routine practice, with vascular access and procedural technique determined by the operator. The same three cardiologists assessed baseline and final TIMI flow and final MBG. Laboratory data were evaluated by different cardiologists, and the angiographic reviewers were unaware of the laboratory results at the time of grading. Cases with disagreement were reviewed together until a consensus grade was reached. The culprit vessel, thrombus grade, use of predilatation or postdilatation, and thrombus aspiration were taken from the angiograms and procedural records. TIMI flow was graded as 0 for no antegrade passage, 1 for penetration without full distal filling, 2 for delayed but complete filling or clearance, and 3 for normal filling and clearance. Only the consensus TIMI and MBGs were preserved in the analysis dataset; separate reader scores were not available for agreement analysis.

Grade 5 thrombus was treated as evidence of complete occlusion, not as a direct measurement of thrombus volume. We could not establish from the extract whether thrombus grading had been repeated after initial flow was restored. Nor could we reliably determine the timing or indication for aspiration, antithrombotic treatment, or intracoronary rescue medication. These treatments were not included as if they were baseline patient characteristics.

### 2.4. Assessment of Myocardial Blush Grade and Definition of No-Reflow

Myocardial blush grade (MBG) on the final angiogram ranged from absent blush or persistent staining (grade 0), through minimal blush (1) and diminished but visible blush (2), to blush comparable to an uninvolved territory (3). We classified an event as no-reflow when impaired reperfusion was documented without significant residual stenosis, dissection, epicardial spasm, or another evident mechanical explanation. Residual stenosis, dissection, and epicardial spasm were assessed during review of the available angiograms, rather than inferred solely from procedural reports. The endpoint covered final TIMI ≤ 2, final TIMI 3 with MBG 0–1, and recorded intraprocedural no-reflow even if flow and blush later recovered. Patients without a recorded event who had final TIMI 3 and MBG ≥ 2 were classified as reflow. The registry did not preserve separate records of each mechanical exclusion step or complete time-stamped angiographic sequences to verify every transient event retrospectively.

For sensitivity analyses based strictly on the final angiogram, we defined impaired reperfusion as final TIMI ≤ 2 or final TIMI 3 with MBG 0–1. Patients recorded as having no-reflow but ending with TIMI 3/MBG ≥ 2 were handled as recovered-flow cases. This classification describes the recorded final angiogram; it does not establish how long any earlier flow disturbance lasted or what caused it.

### 2.5. Study Endpoints

We examined whether admission CALLY was related to angiographic no-reflow during or after primary PCI. We also assessed how well CALLY distinguished patients with and without the event. For the sensitivity analyses, the endpoint was limited to impaired perfusion on the final angiogram; patients whose final flow had recovered were either counted as non-events or left out of the analysis.

### 2.6. Statistical Analysis

Statistical work was performed in Python 3.12.14 with NumPy 2.3.5, SciPy 1.17.0, pandas 2.2.3, scikit-learn 1.8.0, and Matplotlib 3.10.8. We report continuous variables as medians (Q1–Q3), comparing groups with two-sided Mann–Whitney U tests. Categorical comparisons used Pearson’s chi-square test when all expected cell counts were at least five, Fisher’s exact test for smaller 2 × 2 tables, and the Fisher–Freeman–Halton test with 200,000 Monte Carlo samples for larger sparse tables. There were no missing observations in the 1245-patient analytic sample. Our initial logistic model included ln(CALLY), age, sex, hypertension, diabetes, and smoking. During revision, we added baseline TIMI category, culprit artery, grade 5 thrombus, Killip ≥ II at PCI start, and door-to-balloon time in 10-min increments. TIMI 0 and RCA were the reference categories, and LMCA was grouped with ramus intermedius because of low numbers. Clinical relevance guided variable selection; it was not based on univariable significance tests. The expanded model and sensitivity analyses were undertaken after the initial analysis.

We estimated logistic coefficients by maximum likelihood using Newton–Raphson iterations implemented with NumPy and SciPy linear algebra, including step halving and convergence checks. Wald confidence intervals were derived from the inverse observed information matrix. As a cross-check, we fitted the expanded model using unpenalized logistic regression in scikit-learn. Effects are shown per unit of ln(CALLY), per 10 years of age, or for the categorical comparison specified. The effect of doubling CALLY was calculated from its log coefficient multiplied by ln(2). For continuous predictors, restricted cubic splines used knots at the 10th, 50th, and 90th percentiles; nested likelihood-ratio tests assessed evidence of nonlinearity. We did not put CALLY and its constituent laboratory measures into the same model. Nor did we treat final TIMI/MBG or treatments potentially given after no-reflow as baseline predictors.

We checked the stability of the association in several ways: by changing the angiographic adjustment variables and endpoint definition, restricting the baseline TIMI or Killip groups, and adding eGFR and admission hemodynamic measures. To examine possible effects of unusually low CRP and extreme CALLY observations, we used empirical data restrictions, winsorization and trimming at the 1st and 99th percentiles, approximate GLM Cook and DFBETA measures, and refitting after the removal of each patient in turn. Influence diagnostics were used to explore sensitivity to groups of observations. Flagged records were not assumed to be erroneous and were retained in the primary analysis.

Every single-marker ROC comparison was based on the same 1245 patients. Lower values were treated as the adverse direction for CALLY, albumin, lymphocytes, and PNI; for CRP, CAR, NLR, SII, PLR, and SIRI, higher values were considered adverse. We calculated DeLong confidence intervals and paired comparisons from empirical placement values, assigning half credit to ties. Holm correction was applied to the nine comparisons involving CALLY. Wilson intervals were calculated for sensitivity, specificity, and predictive values at the Youden-selected cutpoint. Likelihood-ratio tests compared a model with separate log-transformed CRP, albumin, and lymphocyte terms against the fixed CALLY combination; a second comparison assessed CAR with and without log lymphocytes.

For internal validation, we drew 1000 ordinary bootstrap samples and refitted models with and without CALLY each time. These resamples were used to estimate optimism in the AUC, Brier score, and calibration slope. We also reselected the Youden threshold within each sample to assess how much the apparent operating characteristics depended on cutoff selection. This is internal resampling, not external validation. Secondary tests were exploratory and generally not adjusted for multiple testing. All tests were two-sided, with *p* < 0.05 used as the conventional significance level; the random seed was 20261025. Numerical calculations were performed in the Python environment specified above. AI-assisted tools were used only for language editing and grammar improvement. The authors take full responsibility for the scientific content, data interpretation, and conclusions of the manuscript.

## 3. Results

Among 2756 screened admissions, 1511 were excluded and 1245 were eligible for analysis. Angiographic no-reflow was recorded in 206 patients (16.5%), leaving 1039 (83.5%) in the reflow group (Figure 1). Patients with no-reflow were older (median 62.0 (54.3–71.8) vs. 58.0 (51.0–67.0) years; *p* < 0.001) and had more hypertension (46.6% vs. 38.0%; *p* = 0.021) and cerebrovascular disease (4.4% vs. 1.3%; *p* = 0.005). Current or former smoking was less common (62.6% vs. 70.3%; *p* = 0.030), whereas heart rate was slightly higher (78 C vs. 75 (65–85) beats/min; *p* = 0.009). The other baseline measures listed in Table 1—including sex, diabetes, family history, previous CAD, hyperlipidemia, BMI, and admission blood pressure—did not differ significantly.

The angiograms showed clear differences in culprit artery distribution, pre-PCI TIMI flow, and thrombus grade (*p* = 0.002, *p* < 0.001, and *p* < 0.001, respectively). Initial TIMI 0 flow was found in 79.6% of the no-reflow group and 65.0% of the reflow group. Grade 5 thrombus was recorded in 81.1% and 67.0%, respectively. Aspiration was also more common with no-reflow (10.2% vs. 2.3%; *p* < 0.001). MI location and use of predilatation or postdilatation were not significantly different (Table 1).

At PCI start, Killip class was II or higher in 14 of 206 patients with no-reflow (6.8%) and 45 of 1039 with reflow (4.3%; *p* = 0.128). The test across all four Killip categories gave *p* = 0.073. Median door-to-balloon time was 15 (13–19) minutes in the no-reflow group and 15 (13–17) minutes in the reflow group (*p* = 0.170). Of the 147 patients presenting with TIMI 3 flow, two had recorded no-reflow; Table A1 provides their descriptive comparison.

Compared with the reflow group, patients with no-reflow had higher urea, uric acid, and CRP and lower albumin, hemoglobin, and lymphocyte counts (all *p* < 0.05). Creatinine and the other reported blood-cell counts did not differ significantly. Admission eGFR was lower in patients with no-reflow (90.8 (72.6–104.8) vs. 97.5 (81.9–106.0) mL/min/1.73 m^2^; *p* < 0.001). Median CALLY was 1.11 (0.39–1.92) versus 1.33 (0.73–2.11) (*p* < 0.001); the corresponding ln(CALLY) medians were 0.11 (−0.94–0.65) and 0.28 (−0.32–0.75) (*p* < 0.001) (Table 2).

**Table 2:** Laboratory variables and CALLY according to no-reflow status.

Variable	Reflow (n = 1039)	No-Reflow (n = 206)	*p*-Value
Laboratory variables			
Urea, mg/dL	31.0 (25.0–38.0)	35.0 (27.3–43.0)	<0.001
Creatinine, mg/dL	0.85 (0.73–1.00)	0.86 (0.76–1.02)	0.169
Admission eGFR, mL/min/1.73 m^2^	97.5 (81.9–106.0)	90.8 (72.6–104.8)	<0.001
Uric acid, mg/dL	5.20 (4.40–6.20)	5.60 (4.73–6.60)	<0.001
CRP, mg/L	3.66 (3.22–9.02)	4.17 (3.22–14.55)	0.016
Albumin, g/L	38.70 (36.60–41.00)	37.60 (34.83–40.10)	<0.001
WBC count, ×10^9^/L	11.30 (9.18–14.06)	11.54 (9.09–14.15)	0.698
Hemoglobin, g/dL	14.10 (12.80–15.20)	13.80 (12.20–14.78)	0.015
Platelet count, ×10^9^/L	231.0 (195.0–271.0)	228.5 (188.8–277.0)	0.601
Neutrophil count, ×10^9^/L	8.76 (6.71–11.25)	8.82 (6.72–11.41)	0.644
Lymphocyte count, ×10^9^/L	1.65 (1.19–2.24)	1.57 (1.06–2.11)	0.030
Monocyte count, ×10^9^/L	0.52 (0.39–0.69)	0.54 (0.38–0.74)	0.269
CALLY index			
CALLY index	1.33 (0.73–2.11)	1.11 (0.39–1.92)	<0.001
ln(CALLY)	0.28 (−0.32–0.75)	0.11 (−0.94–0.65)	<0.001

**Figure 2:**
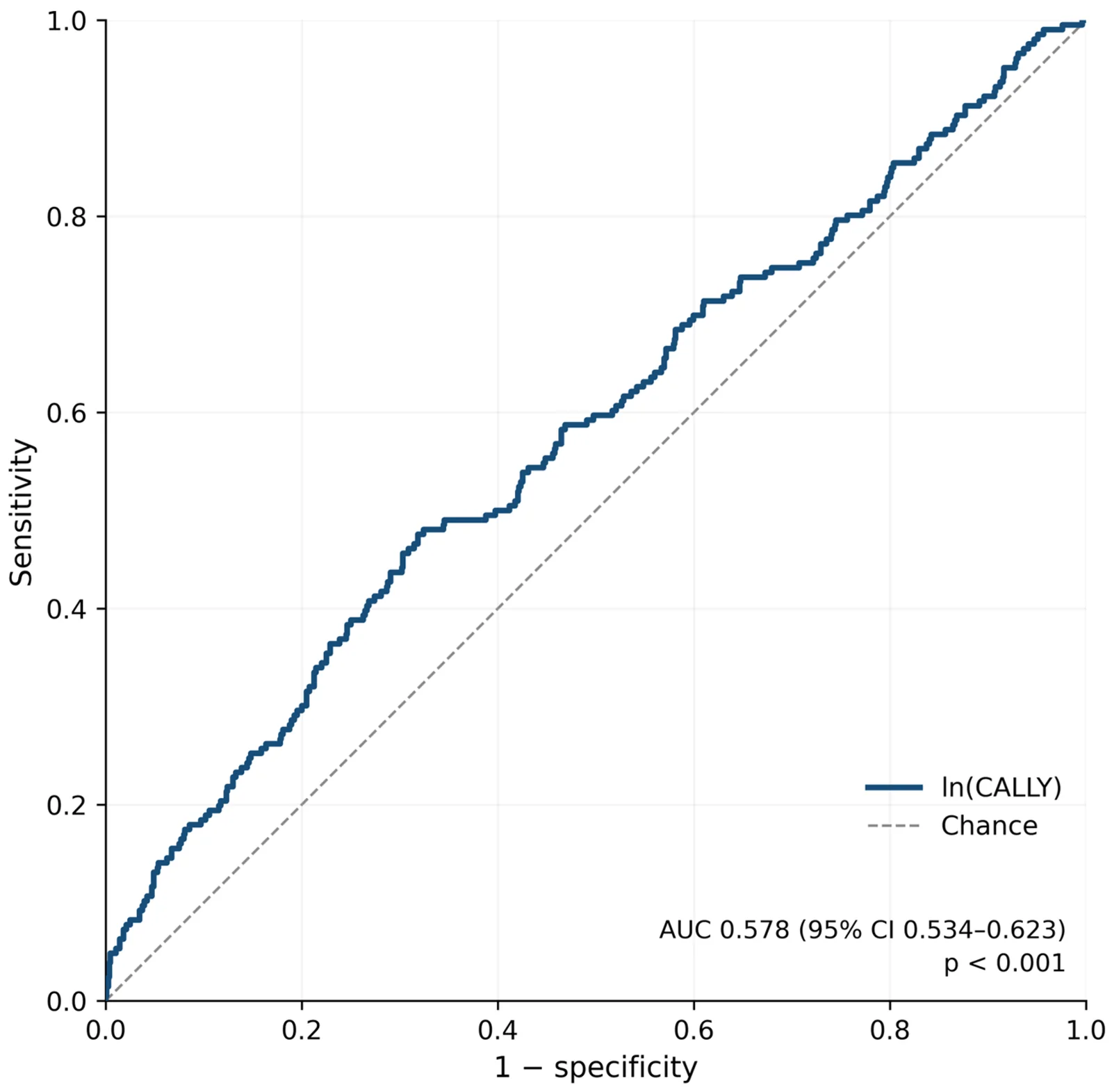
CALLY ROC curve for angiographic no-reflow in 1245 patients, including 206 events. Lower CALLY indicates the adverse direction. AUC = 0.578 (95% CI 0.534–0.623); the diagonal denotes chance discrimination. AUC, area under the curve; CI, confidence interval; ROC, receiver operating characteristic.

Values are median (Q1–Q3). All comparisons used the two-sided Mann–Whitney U test. ln(CALLY) denotes the natural logarithm. CALLY = albumin (g/L) × lymphocytes (×10^9^/L)/[CRP (mg/L) × 10]. Raw and logarithmic CALLY yield identical rank-based *p*-values. Abbreviations: CALLY, C-reactive protein–albumin–lymphocyte index; CRP, C-reactive protein; WBC, white blood cell. On its own, CALLY distinguished no-reflow poorly (AUC 0.578, 95% CI 0.534–0.623; *p* < 0.001). At the displayed Youden threshold of CALLY ≤0.905, equivalent to ln(CALLY) ≤−0.100, 98 of 206 events were identified, while 708 of 1039 patients without the event were classified correctly. Sensitivity was 47.6% and specificity 68.1%. Positive predictive value was 22.8%, and negative predictive value was 86.8% (Table 3; Figure 2).

**Table 3:** ROC analysis of CALLY for no-reflow.

Parameter	Estimate
AUC (95% CI)	0.578 (0.534–0.623)
*p*-value versus AUC = 0.5	<0.001
Displayed ln(CALLY) cut-off	≤−0.100
Equivalent displayed CALLY cut-off	≤0.905
Sensitivity, % (95% CI)	47.6 (40.9–54.4)
Specificity, % (95% CI)	68.1 (65.2–70.9)
Positive predictive value, % (95% CI)	22.8 (19.1–27.0)
Negative predictive value, % (95% CI)	86.8 (84.3–88.9)

DeLong estimates describe the AUC and its 95% CI, with lower CALLY indicating the adverse direction. Sensitivity, specificity, and predictive-value intervals use the Wilson method. ROC ordering is unchanged by the logarithm. The two rounded cutoffs shown in Table 3 refer to the same point on the empirical ROC curve. The unrounded CALLY value was 0.904043126684636. In this dataset, classifying CALLY as ≤0.905 or ln(CALLY) as ≤−0.100 gives identical results. Calculations used the unrounded value. The reported Wilson intervals are conditional on that choice and do not capture uncertainty from selecting the cutoff. Threshold selection was repeated in bootstrap samples (Appendix A), but no independent cohort was available to validate it. Abbreviations: AUC, area under the curve; CALLY, C-reactive protein–albumin–lymphocyte index; CI, confidence interval; ROC, receiver operating characteristic.

**Figure A1:**
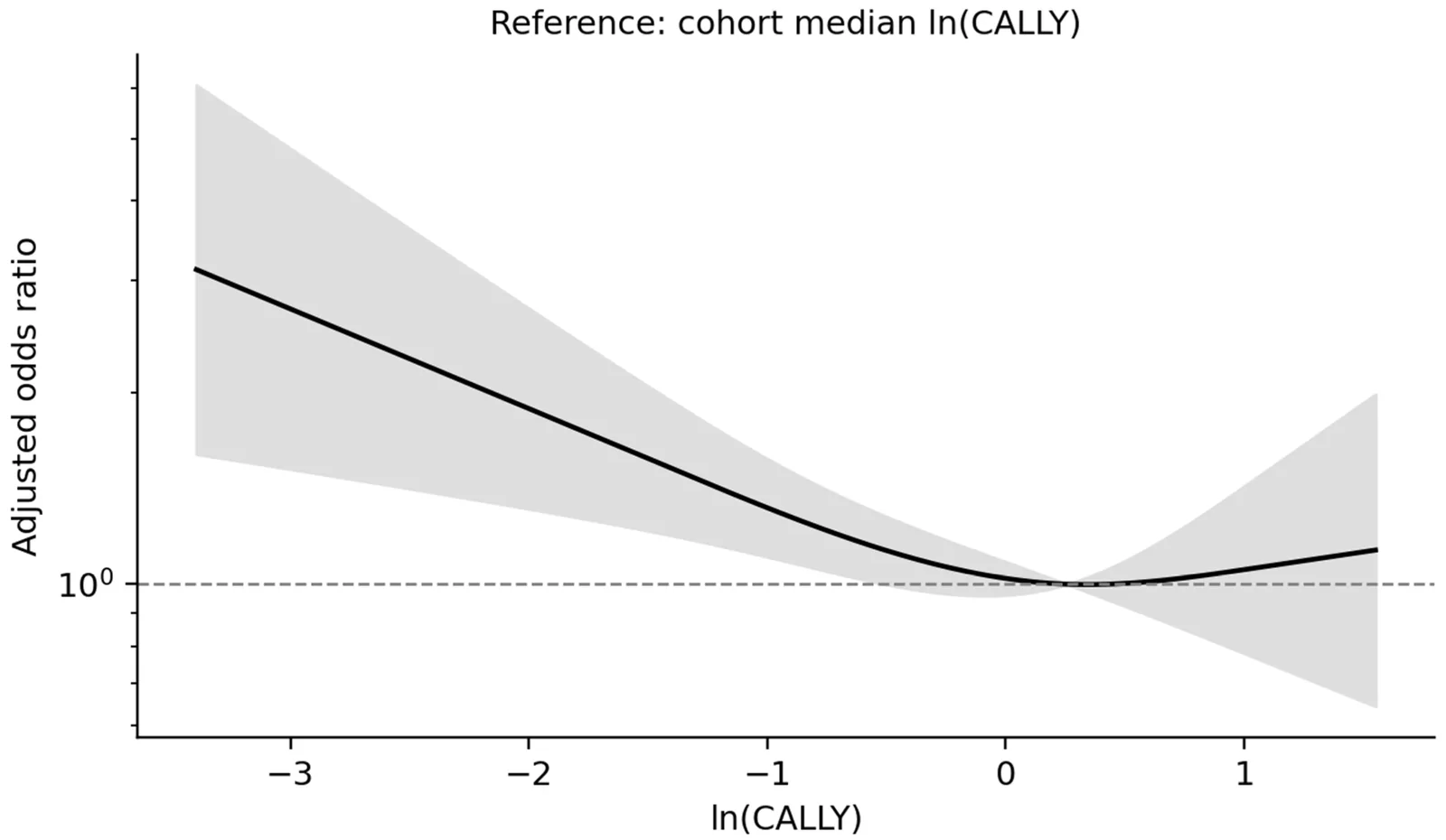
Expanded-model restricted cubic spline for ln(CALLY), with 95% confidence band and the cohort median as reference. Nonlinearity *p* = 0.135; the test does not prove a linear relationship.

The association with ln(CALLY) persisted after the additional angiographic and clinical variables were included. Its OR changed from 0.778 (95% CI 0.679–0.892; *p* < 0.001) in the original clinical model to 0.800 (0.693–0.923; *p* = 0.0023) in the expanded model. This corresponds to an OR of 0.857 (0.776–0.946) for a doubling of CALLY. Age was also associated with no-reflow (OR 1.237 per 10 years, 1.055–1.451; *p* = 0.009) (Table 4). Spline tests did not detect departure from linear log-odds for ln(CALLY), age, or door-to-balloon time (*p* = 0.135, 0.165, and 0.885). These tests do not establish that the relationships are strictly linear (Figure A1).

The endpoint could be divided into 179 cases with final TIMI ≤2, six more with final TIMI 3 and MBG 0–1, and 21 recorded no-reflow events in which final flow and blush had recovered. Of 74 patients with MBG 0–1, 68 also had final TIMI ≤2. When only the 185 patients with impaired final reperfusion were counted as events, the adjusted ln(CALLY) OR was 0.832 (0.717–0.965; *p* = 0.015). Removing the 21 recovered-flow patients altogether gave an OR of 0.816 (0.702–0.948; *p* = 0.008). Associations were similar in patients presenting with baseline TIMI 0–2 or TIMI 0 alone (Table 5).

In the paired ROC comparisons, CALLY was not significantly better than CRP, albumin, lymphocytes, CAR, NLR, SII, PLR, SIRI, or PNI (Table 6). Allowing the three CALLY components to have separate coefficients improved model fit compared with the fixed composite (LR = 10.71, df = 2; *p* = 0.0047). In contrast, adding lymphocytes to a CAR model did not improve fit (*p* = 0.801). The corresponding adjusted-marker analyses appear in Table A2.

**Figure A2:**
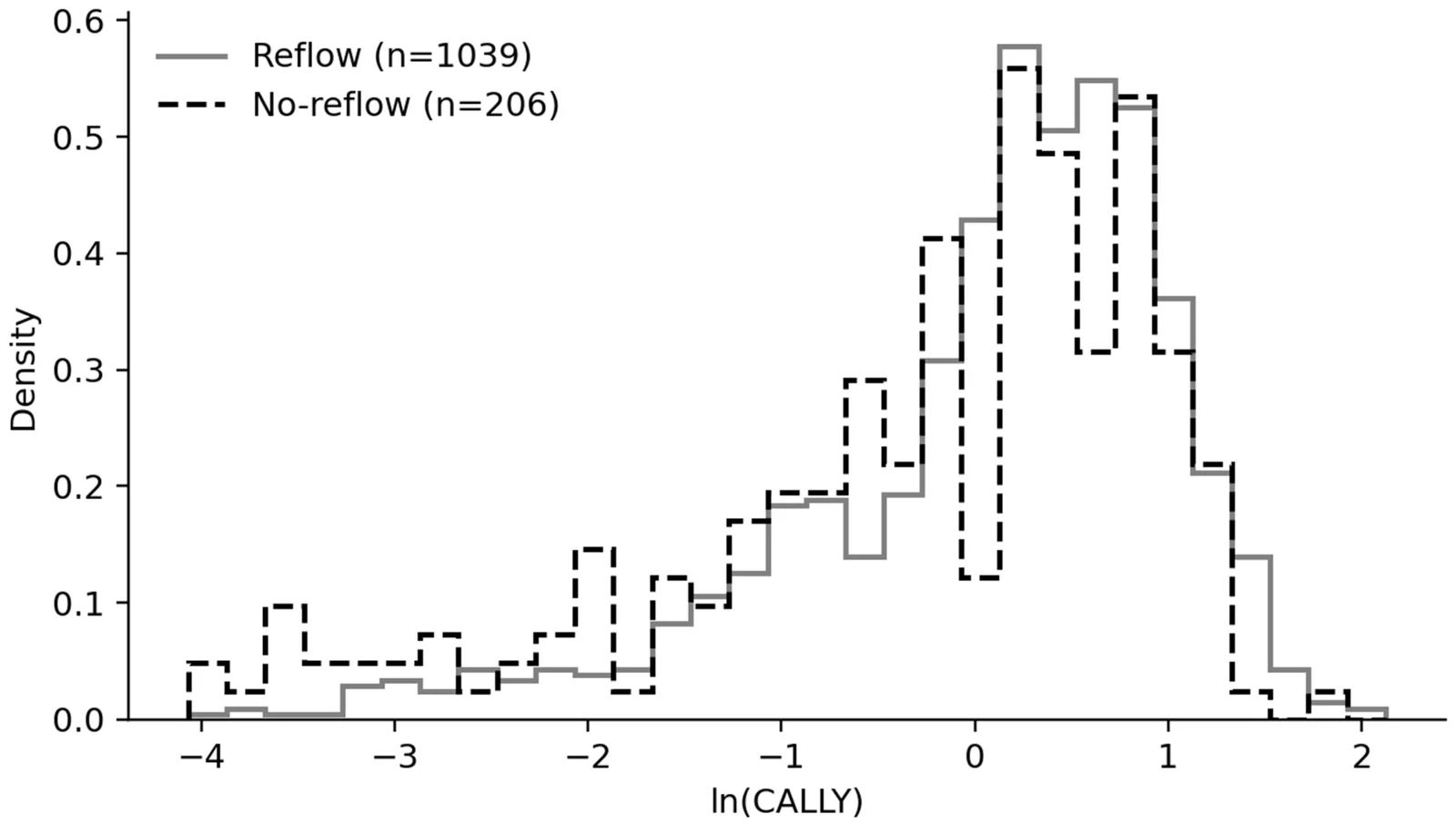
Distribution of ln(CALLY) in the two recorded outcome groups, illustrating substantial overlap.

The result was little changed by winsorizing the extreme ln(CALLY) values. Removing both 1% tails, however, weakened the association (OR 0.858, 95% CI 0.730–1.008; *p* = 0.063). The DFBETA exclusion analysis removed 92 patients, including 71 of the 206 events, and gave an OR of 0.844 (95% CI 0.670–1.063; *p* = 0.149) (Table A3). Leaving out patients one at a time produced ORs from 0.792 to 0.813 (highest *p* = 0.0050), suggesting that no single observation drove the finding. Exactly 287 patients had CRP of 3.22 mg/L; after excluding them, the OR was 0.769 (0.656–0.901; *p* = 0.0012). Further checks are shown in Table A3, with the distributions in Figure A2.

Adding CALLY changed the apparent AUC from 0.715 to 0.723 and the optimism-corrected AUC from 0.691 to 0.699 (corrected difference 0.0077). The corrected calibration slope was 0.764. Although a likelihood-ratio test favored the model containing CALLY (*p* = 0.0026), the difference in discrimination was small (Table A4). With the threshold reselected in each bootstrap sample, corrected sensitivity and specificity were 45.7% and 67.8%; PPV and NPV were 21.9% and 86.3%.

## 4. Discussion

We found an association between lower admission CALLY and recorded angiographic no-reflow in patients undergoing primary PCI for STEMI. It remained after adjustment for the measured clinical and angiographic variables, including baseline flow, culprit artery, grade 5 thrombus, Killip class, and door-to-balloon time. The clinical interpretation is less straightforward. CALLY distinguished the outcome poorly (AUC 0.578), and adding it to the expanded model increased the optimism-corrected AUC by only 0.0077. Nor was the index demonstrably better than the individual measurements or simpler composite markers.

The analysis restricted to impaired reperfusion on the final angiogram also showed an association with CALLY (185 events; adjusted OR 0.832, 95% CI 0.717–0.965). This finding is more directly relevant to the final angiographic result. The 21 recorded events with recovered final flow could not be independently verified from complete procedural sequences, and their clinical significance cannot be assumed to match that of impaired final reperfusion.

A patent epicardial artery does not necessarily mean that the microcirculation has recovered. Distal embolization, ischemic injury, and reperfusion-related processes may act together, with edema, vasoconstriction, oxidative injury, and leukocyte interactions contributing to persistent impairment of tissue flow [13]. A blood test at admission may reflect these processes, but it cannot identify which one is responsible for an individual episode of no-reflow.

Interpretation of the components also requires care. CRP changes with inflammation, whereas albumin and lymphocyte counts are influenced by acute illness and a range of other factors; lymphocyte regulation, in particular, extends well beyond ischemic injury [14]. Samples in this study were taken after symptom onset, but the interval from symptom onset to sampling was unavailable. Thus, a low CALLY value could be a consequence of a larger or longer evolving infarction rather than evidence of vulnerability that preceded it. One albumin measurement during an acute STEMI admission is not evidence of chronic malnutrition.

Fedai and colleagues studied 1785 patients with STEMI using an XGBoost model and defined no-reflow by failure to achieve TIMI 3 flow [11]. Our study addresses a related question in a different dataset, providing adjusted effect estimates and considering additional angiographic endpoint definitions. It does not validate the Fedai model. The regression approach itself is not the distinguishing contribution; the value here lies in testing how much of the CALLY association remains after broader adjustment and in showing how little discrimination the index provides.

There is also a relationship to the previous CALLY study of contrast-associated acute kidney injury (CA-AKI) conducted at the same hospital [12]. That study covered January 2018 to December 2024; the present cohort spans January 2024 to January 2026. Records for the current analysis were extracted afresh under a separate ethics approval and were not drawn from the earlier research database. Nevertheless, patients treated during the overlapping year could appear in both cohorts. We have not measured that overlap and cannot regard the samples as entirely independent.

Earlier reports have linked inflammatory-cell indices with poor reperfusion after primary PCI [6,15,16,17,18]. Between-study differences in laboratory timing, patient selection, adjustment, and the definition of no-reflow complicate comparisons. In our own data, the three components modeled separately fitted better than CALLY’s fixed weighting. Adding lymphocytes to CAR, however, contributed little. Although exploratory, these observations question whether each CALLY component provides useful independent information for this endpoint.

Baseline TIMI flow and grade 5 thrombus were strongly correlated. The reversal of the grade-5 coefficient when both were included should not be interpreted biologically. This combined model was used to examine the stability of the CALLY association, supported by models that included either angiographic variable separately.

Albumin deserves particular attention because it is affected by both inflammation and the patient’s wider physiological condition. Albumin-related indices have been associated with no-reflow in other cohorts [8,19,20], but the study populations and cutpoints vary. We found no statistically significant AUC advantage for CALLY over albumin or CAR. Albumin’s numerically higher AUC should not be taken as proof that it performs better.

The inflammatory changes accompanying myocardial infarction occur alongside metabolic disturbances. Prior work has examined uric acid-based measures and metabolic indices in relation to impaired reperfusion [21,22,23]. These associations place CALLY in a wider group of admission markers of systemic illness, rather than establishing a distinct mechanism for the composite.

Nutritional scores have also been investigated in acute coronary syndrome cohorts [9,24]. Their use does not make CALLY a validated measure of nutritional status. In our dataset, distributions of CALLY and PNI overlapped considerably between patients with and without no-reflow, and the difference between their AUCs was not significant.

All three CALLY components are routinely measured, which makes the index easy to calculate. That convenience should not be confused with clinical accuracy. At the chosen threshold, 108 of the 206 no-reflow events would have been missed, and only 22.8% of patients testing positive had the event. Moreover, the increase in optimism-corrected model AUC was very small, and estimating the three components separately improved fit compared with their fixed combination in CALLY. These findings do not justify a bedside classification threshold; they support further examination of an observed association. The observed association does not show that modifying CRP, albumin, or lymphocyte count would improve reperfusion outcomes.

The sensitivity analyses also place limits on the stability of the estimate. Trimming the upper and lower 1% of ln(CALLY) values attenuated the OR from 0.800 to 0.858, with a confidence interval that included no association (95% CI 0.730–1.008; *p* = 0.063). Excluding DFBETA-flagged observations gave an OR of 0.844 (95% CI 0.670–1.063). That analysis removed 71 of 206 events and substantially changed the population. It is an influence diagnostic, not an estimate from a corrected dataset. The flagged observations are not necessarily invalid. Although no single patient drove the primary result, exclusion of groups of observations showed greater uncertainty than the primary model alone suggests.

### Limitations

This study has several limitations. Its retrospective, single-center design precludes causal inference and leaves room for confounding. We were able to include Killip class at PCI start and door-to-balloon time, but not total ischemic duration or the time from symptom onset to blood sampling. Door-to-balloon time captures in-hospital delay but cannot account for total myocardial ischemic time. Adjustment was therefore limited to the variables actually measured, with residual confounding by ischemic duration and acute-event severity still possible. The admission laboratory results may partly reflect the ongoing infarction. Repeat measurements were unavailable, preventing assessment of how CALLY changed over time.

Selection is another concern: more than half of the screened cases were excluded, commonly because laboratory or angiographic information was incomplete. Missingness may have been related to clinical urgency, illness severity, or procedural complexity. Selection into the analytic cohort could therefore have biased the observed CALLY–no-reflow association itself, in addition to limiting generalizability. We could not compare excluded and included patients, so the direction and size of any resulting bias are unknown.

History variables were taken from routine records and may have been misclassified. In particular, the low recorded prevalence of hyperlipidemia may reflect incomplete documentation; an undocumented history could not be distinguished from a negative entry. Such errors may leave residual confounding by cardiovascular risk factors. Smoking combined current and former use, so adjustment for this variable does not represent adjustment for active tobacco exposure. Differences in smoking status and exposure could not be resolved. Likewise, admission creatinine alone cannot describe a patient’s established renal function. The incomplete disease-specific definitions for the record-based exclusions also limit reproducibility of screening.

Our outcome combined final angiographic abnormalities with events recorded during PCI. Separate reader grades and complete procedural timelines were not retained, limiting assessment of reader agreement and of recovered-flow events. Mechanical explanations for impaired flow were assessed from the available angiograms, but itemized records of these exclusions were not retained, limiting independent verification of endpoint specificity. The stage of thrombus grading and timing of rescue therapy were also uncertain. Two entries showed final TIMI 0 with MBG 3, and two showed baseline TIMI 3 with thrombus grade 5; they were left as recorded. Excluding the two final TIMI 0/MBG 3 entries did not materially alter the CALLY estimate. We had no CMR, myocardial contrast echocardiography, or invasive microvascular measurements to confirm microvascular obstruction directly.

We could not check assay-specific CRP reporting limits across the study period or establish the reason for the 3.22 mg/L cluster. Because CRP is in the denominator of CALLY, differences in measurement or reporting practices may alter the index distribution. The displayed CALLY threshold may therefore not transfer directly to other laboratories. The association became weaker after trimming extreme CALLY values, and the additional models were chosen during revision rather than before the study. Many secondary comparisons were exploratory. Bootstrap correction addressed the fitted models but did not account for every analysis choice. Independent replication is therefore needed.

## 5. Conclusions

In this single-center cohort of patients with STEMI, admission CALLY was associated with recorded angiographic no-reflow after adjustment for the clinical and angiographic measures available to us. The index nevertheless discriminated poorly, added little to model performance, and was sensitive to exclusion of extreme values. Studies with standardized adjudication and reliable ischemic-time data are needed before it can be considered for individual patient classification.

## Figures and Tables

**Table 1:** Characteristics of the study population according to no-reflow status.

Variable	Reflow (n = 1039)	No-Reflow (n = 206)	*p*-Value
Demographic characteristics and cardiovascular risk factors			
Age, years	58.0 (51.0–67.0)	62.0 (54.3–71.8)	<0.001
Female sex, n (%)	192 (18.5)	49 (23.8)	0.078
Hypertension, n (%)	395 (38.0)	96 (46.6)	0.021
Diabetes mellitus, n (%)	277 (26.7)	63 (30.6)	0.248
Current or former smoking, n (%)	730 (70.3)	129 (62.6)	0.030
Family history of CAD, n (%)	172 (16.6)	26 (12.6)	0.159
Previous CAD, n (%)	167 (16.1)	23 (11.2)	0.074
Hyperlipidemia, n (%)	63 (6.1)	15 (7.3)	0.510
Cerebrovascular disease, n (%)	13 (1.3)	9 (4.4)	0.005
BMI, kg/m^2^	27.68 (25.65–30.18)	27.27 (25.21–31.23)	0.782
Systolic BP, mmHg	125 (110–140)	125 (100–140)	0.184
Diastolic BP, mmHg	76 (70–80)	75 (63–80)	0.108
Heart rate, beats/min	75 (65–85)	78 (68–87)	0.009
Killip class at PCI start, n (%)			0.073
Class I	994 (95.7)	192 (93.2)	
Class II	36 (3.5)	13 (6.3)	
Class III	8 (0.8)	0 (0.0)	
Class IV	1 (0.1)	1 (0.5)	
Door-to-balloon time, min	15.0 (13.0–17.0)	15.0 (13.0–19.0)	0.170
Infarct and procedural characteristics			
MI location, n (%)			0.060
Anterior	361 (34.7)	92 (44.7)	
Inferior	614 (59.1)	102 (49.5)	
High lateral	38 (3.7)	7 (3.4)	
Isolated posterior	26 (2.5)	5 (2.4)	
Predilatation, n (%)	985 (94.8)	201 (97.6)	0.087
Postdilatation, n (%)	566 (54.5)	124 (60.2)	0.131
Thrombus aspiration, n (%)	24 (2.3)	21 (10.2)	<0.001
Culprit artery, n (%)			0.002
LMCA	3 (0.3)	1 (0.5)	
LAD	379 (36.5)	94 (45.6)	
LCx	206 (19.8)	21 (10.2)	
RCA	442 (42.5)	86 (41.7)	
Ramus intermedius	9 (0.9)	4 (1.9)	
Pre-PCI TIMI flow, n (%)			<0.001
TIMI 0	675 (65.0)	164 (79.6)	
TIMI 1	71 (6.8)	28 (13.6)	
TIMI 2	148 (14.2)	12 (5.8)	
TIMI 3	145 (14.0)	2 (1.0)	
TIMI thrombus grade, n (%)			<0.001
Grade 0	1 (0.1)	0 (0.0)	
Grade 1	6 (0.6)	0 (0.0)	
Grade 2	30 (2.9)	1 (0.5)	
Grade 3	75 (7.2)	2 (1.0)	
Grade 4	231 (22.2)	36 (17.5)	
Grade 5	696 (67.0)	167 (81.1)	

Values are median (Q1–Q3) or n (%). Smoking combines current and former smoking. Continuous variables were compared with two-sided Mann–Whitney U tests. Categorical comparisons used Pearson chi-square or Fisher exact tests; culprit artery, thrombus grade, and the four-category Killip distribution used Fisher–Freeman–Halton tests with 200,000 Monte Carlo samples. A multi-category *p*-value refers to the overall distribution. Door-to-balloon time was measured in minutes from emergency-department registration to balloon inflation; Killip class was assessed at the start of PCI. Abbreviations: BMI, body mass index; BP, blood pressure; CAD, coronary artery disease; LAD, left anterior descending artery; LCx, left circumflex artery; LMCA, left main coronary artery; MI, myocardial infarction; PCI, percutaneous coronary intervention; RCA, right coronary artery; TIMI, Thrombolysis in Myocardial Infarction.

**Table 4:** Univariable and expanded multivariable logistic regression for angiographic no-reflow.

Variable	Univariable OR (95% CI)	*p*-Value	Expanded Adjusted OR (95% CI)	*p*-Value
ln(CALLY), per 1 unit	0.738 (0.648–0.841)	<0.001	0.800 (0.693–0.923)	0.002
Age, per 10 years	1.324 (1.161–1.509)	<0.001	1.237 (1.055–1.451)	0.009
Female sex	1.377 (0.964–1.967)	0.079	1.080 (0.713–1.634)	0.717
Hypertension	1.423 (1.053–1.923)	0.022	1.274 (0.905–1.794)	0.165
Diabetes mellitus	1.212 (0.874–1.680)	0.249	1.131 (0.781–1.637)	0.514
Current/former smoking	0.709 (0.519–0.969)	0.031	1.028 (0.702–1.504)	0.888
Baseline TIMI 1 vs. 0	1.623 (1.015–2.596)	0.043	1.097 (0.480–2.506)	0.826
Baseline TIMI 2 vs. 0	0.334 (0.181–0.616)	<0.001	0.177 (0.062–0.506)	0.001
Baseline TIMI 3 vs. 0	0.057 (0.014–0.232)	<0.001	0.031 (0.006–0.161)	<0.001
LAD vs. RCA	1.275 (0.923–1.761)	0.141	1.463 (1.041–2.058)	0.028
LCx vs. RCA	0.524 (0.316–0.868)	0.012	0.547 (0.325–0.922)	0.024
LMCA/ramus vs. RCA	2.141 (0.736–6.234)	0.162	2.659 (0.790–8.946)	0.114
Killip ≥II vs. I at PCI start	1.611 (0.867–2.992)	0.131	1.353 (0.692–2.644)	0.377
Door-to-balloon time, per 10 min	1.115 (0.924–1.344)	0.255	1.119 (0.908–1.378)	0.292
Thrombus grade 5 vs. 0–4	2.110 (1.455–3.061)	<0.001	0.573 (0.240–1.368)	0.210

Table 4 gives results for all 15 non-intercept terms of the expanded model, estimated in 1245 patients with 206 events. For the categorical variables, TIMI 0 and RCA served as reference groups; LMCA and ramus intermedius were combined. The univariable analyses used the same reference groups. Wald confidence intervals and *p*-values are reported. A one-unit increase in ln(CALLY) represents roughly a 2.72-fold increase in the original index. Expressed on the original scale, doubling CALLY corresponded to an adjusted OR of 0.857 (95% CI 0.776–0.946; *p* = 0.0023). Grade 5 represents an occluded artery rather than a measured thrombus volume. Its VIF was 7.64, compared with 1.09 for ln(CALLY), prompting separate models with baseline TIMI flow and grade 5 (Table 5). The negative coefficient for grade 5 when both variables are entered should not be interpreted as a protective biological association. Abbreviations: CI, confidence interval; OR, odds ratio.

**Table 5:** Selected sensitivity analyses for ln(CALLY).

Specification	N	Events	Adjusted OR (95% CI)	*p*
Final endpoint; recovered-flow patients are non-events	1245	185	0.832 (0.717–0.965)	0.015
Final endpoint; recovered-flow patients excluded	1224	185	0.816 (0.702–0.948)	0.008
Baseline TIMI 0–2 only	1098	204	0.796 (0.690–0.919)	0.002
Baseline TIMI 0 only	839	164	0.806 (0.685–0.948)	0.009
Grade 5 in place of baseline TIMI	1245	206	0.788 (0.683–0.907)	<0.001
Baseline TIMI, without grade 5	1245	206	0.799 (0.692–0.922)	0.002
Expanded model plus eGFR	1245	206	0.801 (0.693–0.926)	0.003
Plus eGFR, SBP, and heart rate	1245	206	0.818 (0.707–0.947)	0.007
Winsorize ln(CALLY) at 1st/99th percentiles	1245	206	0.802 (0.693–0.928)	0.003
Trim both 1% tails of ln(CALLY)	1219	197	0.858 (0.730–1.008)	0.063

Odds ratios describe a one-unit increase in ln(CALLY). We kept the expanded adjustment variables except when one was constant in a restricted sample or was intentionally replaced. These analyses were performed during revision. The final-angiogram endpoint included 185 cases; additional checks of coding, low CRP, and influential observations are listed in Table A1.

**Table 6:** Paired single-marker ROC comparisons for angiographic no-reflow.

Marker	AUC (95% CI)	Paired *p* vs. CALLY	Holm-Adjusted *p*
CALLY	0.578 (0.534–0.623)	Reference	—
CRP	0.553 (0.508–0.598)	0.111	0.775
Albumin	0.600 (0.556–0.644)	0.381	1.000
Lymphocytes	0.548 (0.504–0.592)	0.184	1.000
CAR	0.569 (0.525–0.613)	0.550	1.000
NLR	0.545 (0.500–0.589)	0.173	1.000
SII	0.532 (0.488–0.576)	0.078	0.698
PLR	0.535 (0.491–0.580)	0.089	0.711
SIRI	0.548 (0.504–0.592)	0.267	1.000
PNI	0.597 (0.553–0.640)	0.360	1.000

The same 1245 observations were used for every marker. Lower values indicated worse status for CALLY, albumin, lymphocytes, and PNI; higher values were adverse for the remaining markers. Paired DeLong tests were used with Holm adjustment for nine CALLY comparisons. A nonsignificant difference should not be interpreted as evidence that two markers are equivalent.

## Data Availability

De-identified data supporting the findings of this study may be made available by the corresponding author upon reasonable request, subject to institutional approval and applicable ethical and privacy restrictions. The data are not publicly available because the source dataset contains potentially identifiable patient information.

## Abbreviations

The following abbreviations are used in this manuscript:

AUC	Area under the curve
BMI	Body mass index
BP	Blood pressure
CAD	Coronary artery disease
CALLY	C-reactive protein–albumin–lymphocyte index
CI	Confidence interval
CKD-EPI	Chronic Kidney Disease Epidemiology Collaboration
CRP	C-reactive protein
eGFR	Estimated glomerular filtration rate
LAD	Left anterior descending artery
LCx	Left circumflex artery
LMCA	Left main coronary artery
MBG	Myocardial blush grade
MI	Myocardial infarction
OR	Odds ratio
PCI	Percutaneous coronary intervention
RCA	Right coronary artery
ROC	Receiver operating characteristic
STEMI	ST-segment elevation myocardial infarction
TIMI	Thrombolysis in Myocardial Infarction
WBC	White blood cell

## Appendix A. Additional Analyses

These exploratory analyses complement the expanded model and sensitivity analyses in Table 4, Table 5 and Table 6.

**Table A1:** Baseline TIMI 3 subset compared descriptively with TIMI 0–2.

Characteristic	Baseline TIMI 0–2	Baseline TIMI 3
Patients	1098	147
No-reflow, n (%)	204 (18.6)	2 (1.4)
Age, years	59.0 (52.0–67.0)	60.0 (50.0–67.0)
CALLY	1.28 (0.60–2.02)	1.41 (0.86–2.33)
Door-to-balloon time, min	15.0 (13.0–17.0)	15.0 (14.0–16.5)
Killip ≥ II at PCI start, n (%)	50 (4.6)	9 (6.1)

Values are median (Q1–Q3) or n (%). This descriptive comparison does not determine spontaneous reperfusion, persistent symptoms, or the indication for PCI in the TIMI 3 subset.

**Table A2:** Alternative markers added separately to the same base model.

Marker and Scale	Adjusted OR (95% CI)	*p*	Apparent Model AUC
ln(CALLY)	0.800 (0.693–0.923)	0.002	0.723
ln(CRP)	1.280 (1.087–1.508)	0.003	0.723
Albumin, per 5 g/L	0.636 (0.508–0.796)	<0.001	0.726
ln(lymphocytes)	0.954 (0.678–1.343)	0.789	0.715
ln(CAR)	1.300 (1.111–1.522)	0.001	0.725
ln(NLR)	1.063 (0.824–1.371)	0.639	0.714
ln(SII)	1.051 (0.834–1.324)	0.676	0.714
ln(PLR)	1.040 (0.755–1.431)	0.812	0.714
ln(SIRI)	1.170 (0.947–1.445)	0.145	0.715
PNI, per 5 points	0.830 (0.713–0.966)	0.016	0.718

Each row replaces CALLY with the indicated marker while retaining the other expanded covariates. These exploratory regression *p*-values are not multiplicity-adjusted. CAR = CRP/albumin; NLR = neutrophils/lymphocytes; SII = platelets × neutrophils/lymphocytes; PLR = platelets/lymphocytes; SIRI = neutrophils × monocytes/lymphocytes; PNI = albumin (g/L) + 5 × lymphocytes (10^9^/L). Components and CALLY were never entered together in one model. In a separate unconstrained component model, ln(CRP), ln(albumin), and ln(lymphocytes) had *p* = 0.080, *p* < 0.001, and *p* = 0.836, respectively. Compared with the constrained ln(CALLY) model, the likelihood-ratio statistic was 10.71 on 2 df (*p* = 0.0047). Adding ln(lymphocytes) to a CAR model did not improve fit (LR = 0.063 on 1 df; *p* = 0.801). These are fit comparisons, not demonstrations of superior out-of-sample prediction.

**Table A3:** Further sensitivity and influence analyses.

Specification	n	Events	ln(CALLY) OR (95% CI)	*p*
Killip I at PCI start only	1186	192	0.785 (0.676–0.913)	0.002
Log-transformed door-to-balloon time	1245	206	0.800 (0.693–0.924)	0.002
Grade 4–5 in place of TIMI	1245	206	0.784 (0.681–0.903)	<0.001
Four-category Killip model	1245	206	Not estimable	—
Killip I, II, and III–IV	1245	206	0.800 (0.693–0.923)	0.002
Exclude CRP exactly 3.22 mg/L	958	162	0.769 (0.656–0.901)	0.001
Restrict CRP to >3.34 mg/L	661	117	0.732 (0.605–0.886)	0.001
Exclude final TIMI 0/MBG 3	1243	204	0.802 (0.695–0.926)	0.003
Exclude Cook-flagged records	1169	135	Not estimable	—
Exclude DFBETA-flagged records	1153	135	0.844 (0.670–1.063)	0.149

The CRP restrictions assess the influence of the exact 3.22 mg/L cluster and low values; 3.34 mg/L is not an assay limit. Four-category Killip and Cook-exclusion models were unstable and are not interpreted. Cook/DFBETA exclusions each removed 71 of 206 events. These are influence diagnostics, not routine data cleaning; flagged records are not necessarily invalid and remain in the primary analysis. Leave-one-out OR range: 0.792–0.813; maximum *p* = 0.0050.

**Table A4:** Bootstrap assessment of the contribution of CALLY.

Metric	Without CALLY	With CALLY
Apparent AUC	0.7146	0.7230
Optimism-corrected AUC	0.6910	0.6986
Apparent Brier score	0.1266	0.1252
Optimism-corrected Brier score	0.1304	0.1294
Corrected calibration slope	0.759	0.764

All 1000 bootstrap samples were completed. Both models were refitted per resample. Corrected ΔAUC = 0.0077; lower Brier score is better. Model fit with versus without CALLY was compared by likelihood-ratio testing (LR = 9.10 on 1 df; *p* = 0.0026), not an ordinary DeLong test of nested fitted model predictions. Reselecting the Youden cutpoint within each bootstrap resample gave optimism-corrected sensitivity 45.7%, specificity 67.8%, PPV 21.9%, and NPV 86.3%. Selected cutpoints had median 0.903 and 2.5th–97.5th percentiles 0.424–1.538. This is a resampling distribution, not a validated clinical threshold interval.
